# Estrogen improved the regeneration of axons after subcortical axon injury via regulation of PI3K/Akt/CDK5/Tau pathway

**DOI:** 10.1002/brb3.1777

**Published:** 2020-08-05

**Authors:** Xiaohui Xia, Changlong Zhou, Xiaochuan Sun, Xuenong He, Chang Liu, Guanyu Wang

**Affiliations:** ^1^ Department of Neurosurgery Yongchuan Hospital Chongqing Medical University Chongqing China; ^2^ Department of Neurosurgery The First Affiliated Hospital of Chongqing Medical University Chongqing China

**Keywords:** CDK5, estrogen, PI3K/Akt, subcortical axonal injury, Tau

## Abstract

**Aim:**

To investigate the effect of estrogen on axon regeneration and neurological recovery after subcortical axon injury, and further explore its underlying molecular mechanisms.

**Method:**

Subcortical axonal fiber injury model was used in this study. Morris water maze was conducted to detect the learning and memory ability of the rats; modified neurological severity score (mNSS) and beam walking test were performed to evaluate the behavioral; and diffusion tensor imaging (DTI) was used for the determination of recovery after subcortical axonal injury, while Western blotting was performed to detect the expression of p‐Akt, CDK5, p‐Ser262, p‐Ser404, and p‐Thr205.

**Results:**

Compared with the Sham group, the injury of subcortical axonal fiber resulted in higher mNSS, higher beam walking scores, longer time of escape latency, less number, time and shorter distance of crossing the quadrant, and less FA values. After ovariectomy, the mNSS, beam walking scores, and escape latency reached the peak; inversely, the others reached a minimum. High estrogen treatment reduced the mNSS, beam walking score, and escape latency; improved the number, time, and distance of crossing the quadrant; and increased the FA value. Western blotting results showed that estrogen increased the expression of p‐Akt and decreased the expression of CDK5, p‐Ser262, p‐Ser404, and p‐Thr205. All the changes were counteracted to some extent by Akt inhibitor LY294002.

**Conclusion:**

After subcortical axonal injury, estrogen could improve the regeneration of axons and improve their functions via regulating the PI3K/Akt/CDK5/Tau pathway.

## Graphical abstract

Axonal injury is a common anatomical and pathological basis for neurological dysfunction caused by many kinds of CNS diseases, such as brain trauma and spontaneous cerebral hemorrhage. The aim of this study was to investigate the effect of estrogen on axon regeneration and neurological recovery after subcortical axon injury, and further explore its underlying molecular mechanisms. The study concluded that after subcortical axonal injury, estrogen could improve the regeneration of axons and improve their functions via regulating the PI3K/Akt/CDK5/Tau pathway.

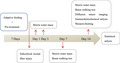

## 1 INTRODUCTION

Axonal injury is a common anatomical and pathological basis for neurological dysfunction caused by many kinds of central nervous system diseases, such as brain trauma (Adams et al., 2018) and spontaneous cerebral hemorrhage (Katsuki & Hijioka, 2017). All of these diseases can cause continuous destruction of axonal fibers in the white matter, leading to neuronal apoptosis, necrosis, axon mutation, and loss of myelin sheath, eventually resulting in irreversible nerve damage and nervous system dysfunction. It is well known that the axons of central nervous system have no regenerative ability in adults, but if the normal brain tissues would establish a new structural connection with the injured tissues after mechanical damage, such as there is a correlation between the injured axons and the normal dendrite, the neurological functions could get a certain extent of restoration (Dancause et al., 2005; Stokowska et al., 2017).

Axonal regeneration after injury is mainly affected by many factors. Among them, Tau phosphorylation is a relatively important factor. They interact with microtubule through repetitive domains and folded domains to stabilize microtubule structure, promote microtubule assembly, and dynamically regulate microtubule stability (Feinstein & Wilson, 2005). After axon injury, Tau phosphorylation occurred, and the sites were mainly at Thr205, Ser262, and Ser404. Excessive phosphorylation of Tau would compete with tubulin to bind with normal Tau protein and other macromolecular tubule‐associated proteins, resulting in the loss of stability of microtubule and nerve fiber degeneration, and affecting axonal regeneration (Lopes et al., 2016). In vivo, the level of Tau protein phosphorylation is dependent on the balance of active protein kinases and phosphatases. Cyclin‐dependent kinase 5 (CDK5) is one of the most important proteins, which phosphorylates Tau protein and reduces its ability of binding with microtubules, alters the morphology of nerve cell, and leads to the disruption of cytoskeleton; thus, the regeneration of axon injury is affected (Xiao et al., 2018; Ye, Fu, & Ip, 2012).

Now that activation of CDK5 is involved in axonal regeneration and plays vital roles (Namgung et al., 2004), a drug targeting the activity of CDK5 would be beneficial for promoting axonal regeneration. It was reported that CDK5 was downstream substrate of PI3K/Akt signaling pathway (Bogush et al., 2007) and estrogen played a key role in regulating PI3K/Akt pathway (Kim et al., 2019; de Oliveira et al., 2018). Furthermore, estrogen promoted the recovery of neurological function and regeneration after axonal injury (Colon & Miranda, 2016; Colon et al., 2016; Xiao et al., 2017); however, the underlying mechanisms were not fully unknown. Therefore, we speculated whether estrogen could affect the activity of CDK5 through PI3K/Akt pathway and inhibit the phosphorylation of Tau protein, thereby promoting the regeneration of axonal injury.

## 2 METHODS AND MATERIALS

### 2.1 Animals

**Figure 1:**
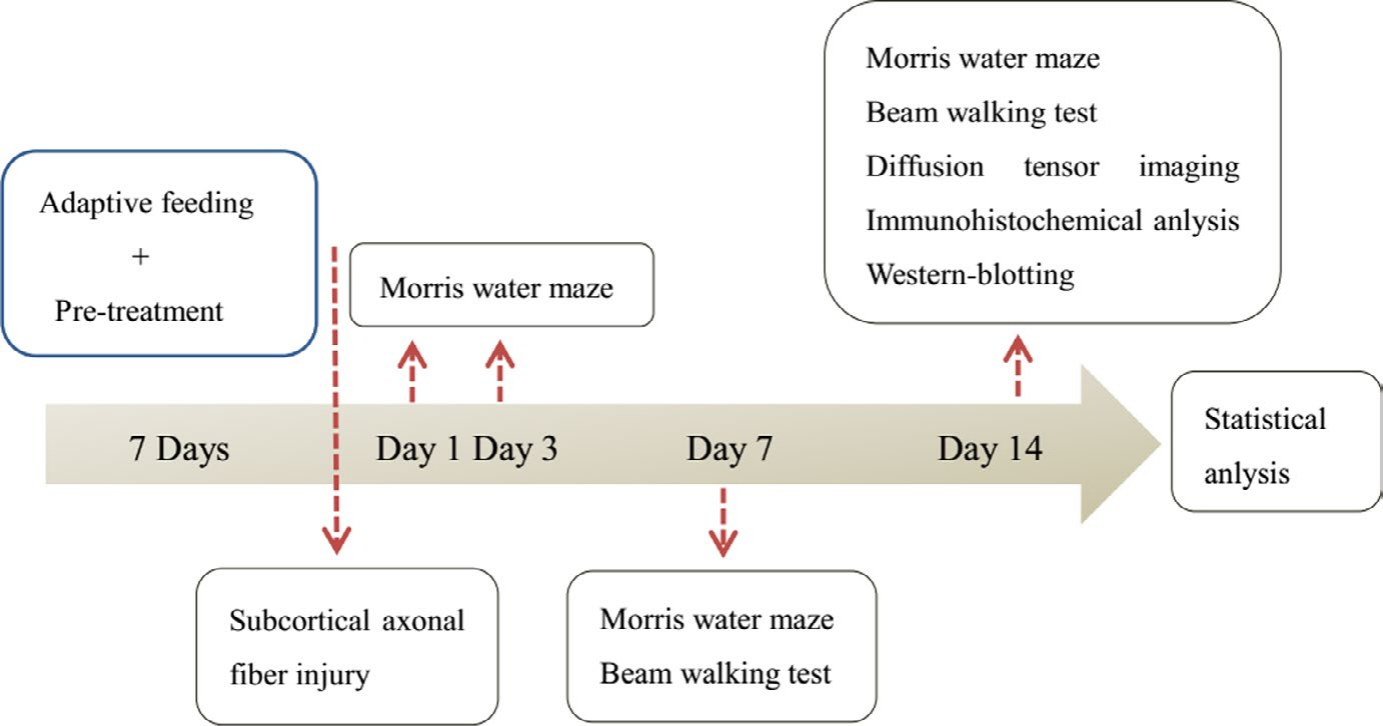
The flowchart of animal experimental procedures

Fifty female Sprague Dawley (*SD*) rats, weighing 220–250 g, provided by Chongqing Medical University Animal Center, were enrolled in the experiments [SCXK (Chongqing) 2016‐0001]. All rats were maintained in the house with a background of temperature 21 ± 1°C and humidity (55 ± 10%). The *SD* rats were randomly divided into five groups: (I) Sham group, the rats received sham operation of scalp incision (Sham, *n* = 10); (II) normal estrogen group, normal feeding (Normal, *n* = 10); (III) Low estrogen group, the rats received a ovariectomy (Low, *n* = 10); (IV) High estrogen group, the rats were fed with estradiol (300 μg/kg) for seven consecutive days before subcortical axonal fiber injury surgery (High, *n* = 10); and (V) LY294002 group, the rats were fed with estradiol and intraventricular injection of LY LY294002 for seven consecutive days before subcortical axonal fiber injury surgery (LY, *n* = 10). All the rats in the latter four groups were received a subcortical axonal fiber injury surgery and continued to be fed according to the preoperative treatment after surgery. The flowchart of animal experimental procedures is shown in Figure 1. This study was approved by the ethics committee of our hospital. All procedures are in strict accordance with the recommendations in the Guide for the Care and Use of Laboratory Animals of the National Institutes of Health.

### 2.2 Ovariectomy

The ovariectomy was kept with the following procedures: First, the rats were anesthetized with 10% chloral hydrate (400 mg/kg) undergoing intraperitoneal injection and then placed in a abdominal decubitus position on a surgery table. An 1‐cm oblique lower incision was made on the lateral side of the dorsal spine, and the skin, subcutaneous tissue, muscle, and peritoneum were incised layer by layer. The ovarian adipose tissue was carefully separated, the periosteum vessels were ligated, the ovaries were removed, and the wound was sutured layer by layer.

### 2.3 LY294002 treatment

PI3K/Akt inhibitor LY294002 was purchased from Sigma‐Aldrich (St. Louis, Mo, USA); LY294002 was injected into the lateral ventricle (20 mM, 5 μL, from the bregma: anteroposterior, −0.8 mm; lateral, 1.5 mm; depth, 3.5 mm) daily for 7 days before axonal fiber injury model establishment (Yin et al., 2015).

### 2.4 Establishment of subcortical axonal fiber injury model

One week after estrogen treatment, the subcortical axonal fiber injury model was established according to the previous report (Dale, Kuang, Wei, & Varon, 1995). In brief, the rats were intraperitoneally anesthetized and fixed on a stereotactic instrument. The scalp was opened, and the anterior fontanelle was exposed. The endocranium was cut longitudinally along the bone window, which was 1 mm near the middle of the anterior fontanelle. The transverse cutting knife was mounted on the electrode clamp of the stereotactic positioner at an angle of 15 degrees to the midsagittal plane and placed on the cerebral cortex in the bone window. Its coordinates were as follows: 0 mm before the front fontanel and 1 mm by the center line. After the cutting knife lowering to the depth 300 µm subcortex, it made a 180 degrees clockwise rotation and then returned to the normal position. The scalp was sutured with a thin layer of gelatin sponge in the bone window.

### 2.5 Morris water maze

Two weeks after axonal fiber injury rats' model establishment and treatment, all the rats were received the Morris water maze test. During the 5 consecutive days of training, each rat was sequentially placed in the water to look for the hidden platform situated in the water. The rats successfully found the platform in 2 min and stayed on it for 10 s; if failed, they were guided to the platform and stayed for 30 s. Each rat was received 4 trials each day, and the interval was longer than 1 hr. At the 6th day, the platform was removed and the same water inlet point was selected. The traveled distance, time, and the number of crossing the target quadrant for 1 min were recorded by the tracking system (Lee, Park, Ahn, & Won, 2016).

### 2.6 Assessments of Neurological function

The modified neurological severity score (mNSS) is a multifunctional evaluation scale that comprises motor, sensory, reflex, and balance tests. The mNSS measurements were performed at day 1, day 3, day 7, and day 14 after axonal fiber injury. The test scale ranges from 0 to 18 (normal score, 0; maximal deficit score, 18; Hsieh et al., 2017).

### 2.7 Beam walking test

The beam walking test was performed on the 7th and 14th day after surgery. For the beam balance component, rats were placed on a 1‐inch‐width beam for 60 s. A normal response is balanced with steady posture for 60 s (a score of 0). Deficits are scored if the rat grasps the side of the beam (a score of 1), hugs the beam and 1 limb falls down from beam (a score of 2), hugs the beam and 2 limb falls off the beam (a score of 3), attempts to balance on beam but falls off from 40 to 59 s (a score of 4), attempts to balance on beam but falls off from 20 to 39 s (a score of 5), or falls off with no attempt to balance or hang on beam in 20 s (a score of 6; Huang et al., 2018).

### 2.8 Diffusion tensor imaging (DTI)

Two weeks after the axon damage model was successfully established, the DTI examination was done. The rats' brain images were acquired using a 7.0‐T ultra‐high field animal MR scanner with aperture 31cm, gradient field strength 290 mT/m, and conversion rate 1160 T m/s. Round ROI was placed in the cortex, outer capsule, hippocampus, corpus callosum, and the corresponding parts of the healthy side around the lesion on the traumatic side. Fractional anisotropy (FA) values of the surrounding area and the corresponding parts of the healthy side were measured, respectively, and the differences in FA values among the groups were analyzed. In the Sham operation group, ROI was placed in the corresponding parts of the bilateral cerebral hemispheres (Lescot et al., 2010; Li et al., 2013).

### 2.9 Immunohistochemical analysis

Two weeks after axonal fiber injury rats' model establishment and treatment, the rats were anesthetized and their limbs were fixed. The chest cavity of the rat was cut open, and a small incision was made at the left apex of the heart. When the indwelling needle was observed to return blood, immediately fixed it. The right auricle was then cut open with ophthalmic scissors, followed by fluid infusion with a micropump. The heart was first perfused with normal saline, and when the color of the liver changed from red to grayish‐white and the color of the liquid from the right atrial appendage was clear, 4% paraformaldehyde could be used for perfusion. During the process of paraformaldehyde perfusion, when the rats were observed to have convulsions in their limbs and stiff and plate‐like bodies, the brain could be extracted. After then, paraffin sections of the brain were made. Briefly, endogenous peroxidase activity within the sections was quenched by incubating the sections with 3% H_2_O_2_ for 10 min after dewaxing and hydration. Tissues were incubated in a humidified chamber with primary antibodies directed against NF200 (1:500). On the following day, the tissues were washed with PBS and incubated with secondary antibody. In the negative controls, the primary antibody was replaced with PBS. They were counterstained with DAB.

### 2.10 Western blotting

Two weeks after axonal fiber injury rats' model establishment and treatment, total proteins from brain tissue were extracted using a total Protein Extraction kit (BestBio Science, China) and the concentrations of the total proteins were determined by using Bioepitope Bicinchoninic Acid Protein Assay kit (Bioworld Technology Co., Ltd, USA). Then, protein samples were separated on polyacrylamide–SDS gels and electroblotted onto nitrocellulose membrane (Millipore, USA). After blocking with TBS, 5% nonfat dry milk for 2 hr, the membrane incubated overnight with primary antibodies: anti‐CDK5, anti‐p‐Tau at Thr205, Ser262, Ser404 sites, anti‐p‐Akt, and β‐actin antibody. After washing in TBST three times, they were incubated with peroxidase‐conjugated antibodies for 45 min at room temperature. The immunoblots were developed using the enhanced chemiluminescence detection system. The band intensity was quantified by ImageJ 1.39u software (National Institutes of Health, NIH, USA).

### 2.11 Statistical analysis

SPSS 19.0 (IBM, New York, USA) was applied to analyze all data. Differences among multiple groups were statistically analyzed using one‐way ANOVA and post hoc comparisons (Dunnett's test). Values of *p* < .05 were considered statistically significant.

## 3 RESULTS

### 3.1 Estrogen improved the memory and cognition function of the rats with ovariectomy after subcortical axonal fiber injury

**Figure 2:**
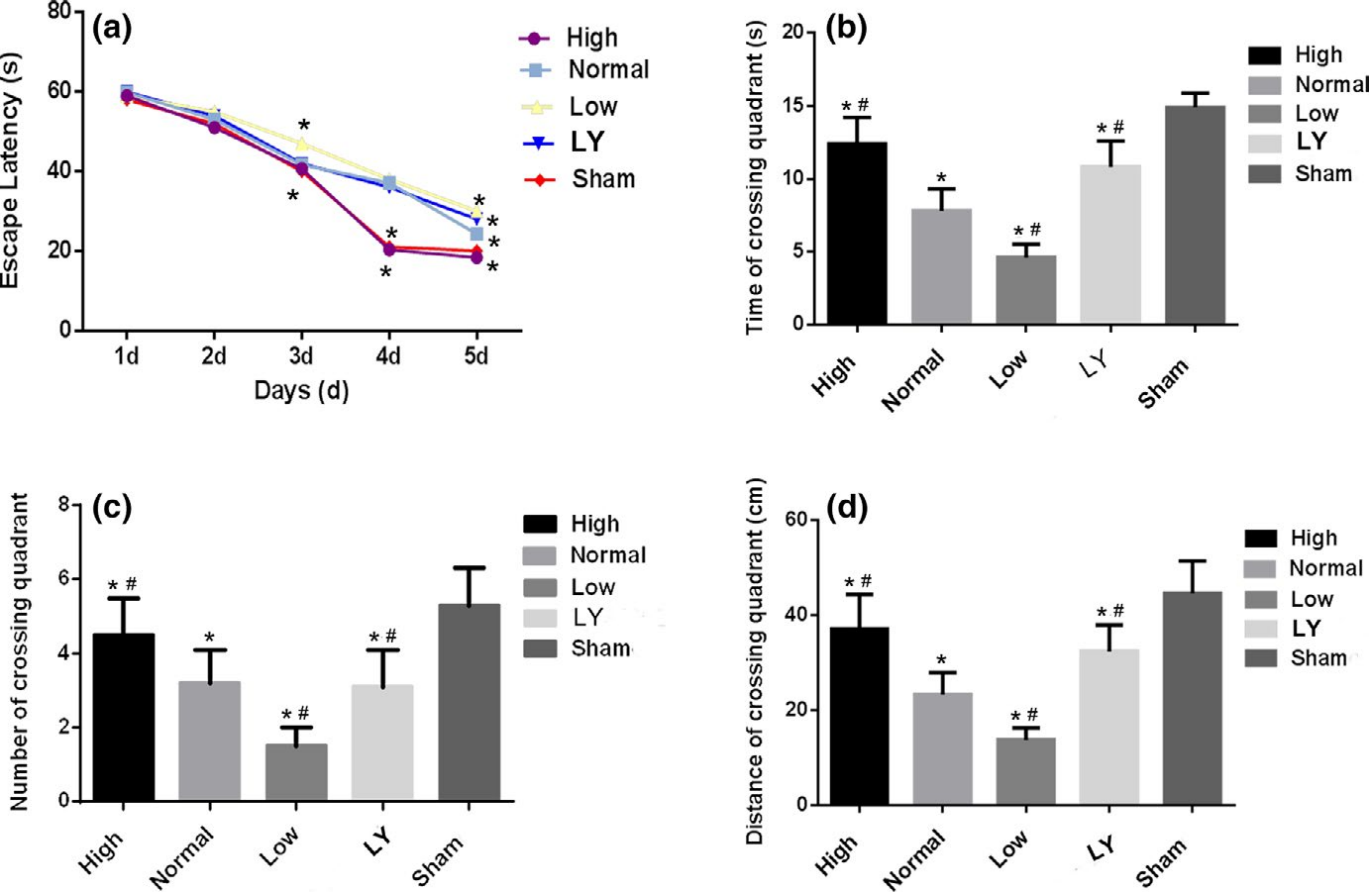
Estrogen improved the memory and cognition function of the rats after subcortical axonal fiber injury. (a) Escape latencies of rats in the water maze test during the five consecutive days. *p < .05 indicated compared with 1st day; (b) the time of crossing target quadrant at 6th day of each group; (c) the numbers of crossing the quadrant of platform at 6th day of each group; (d) the distance traveled by the rats to the hidden platform in the Morris water maze at 6th day of each group; *p < .05 indicated compared with the Sham group; ^#^ *p < *.05 indicated compared with the normal estrogen group. The rats in the Sham group received sham operation of scalp incision as a negative control; in all the other four groups, the rats were received a subcortical axonal fiber injury surgery as the test groups

Morris water maze test was carried out to determine the level of memory and cognition functions. As shown in Figure 2a, during the course of the 5 days' training, there was a day‐to‐day decreased trend of escape latency in each group (*p* < .05). Especially, the decreasing trend was most obvious in the Sham group (*p* < .05). On the 6th day, the ratio of the dwelling time, the number, and the distance of the rats crossing the platform in the quadrant are recorded and presented in Figure 2b–d. Compared to the Sham group, the rats in the other 4 groups spent little time and shorter distance of crossing the quadrant (*p* < .05).

### 3.2 Estrogen treatment improves neurological deficits following subcortical axonal fiber injury

**Figure 3:**
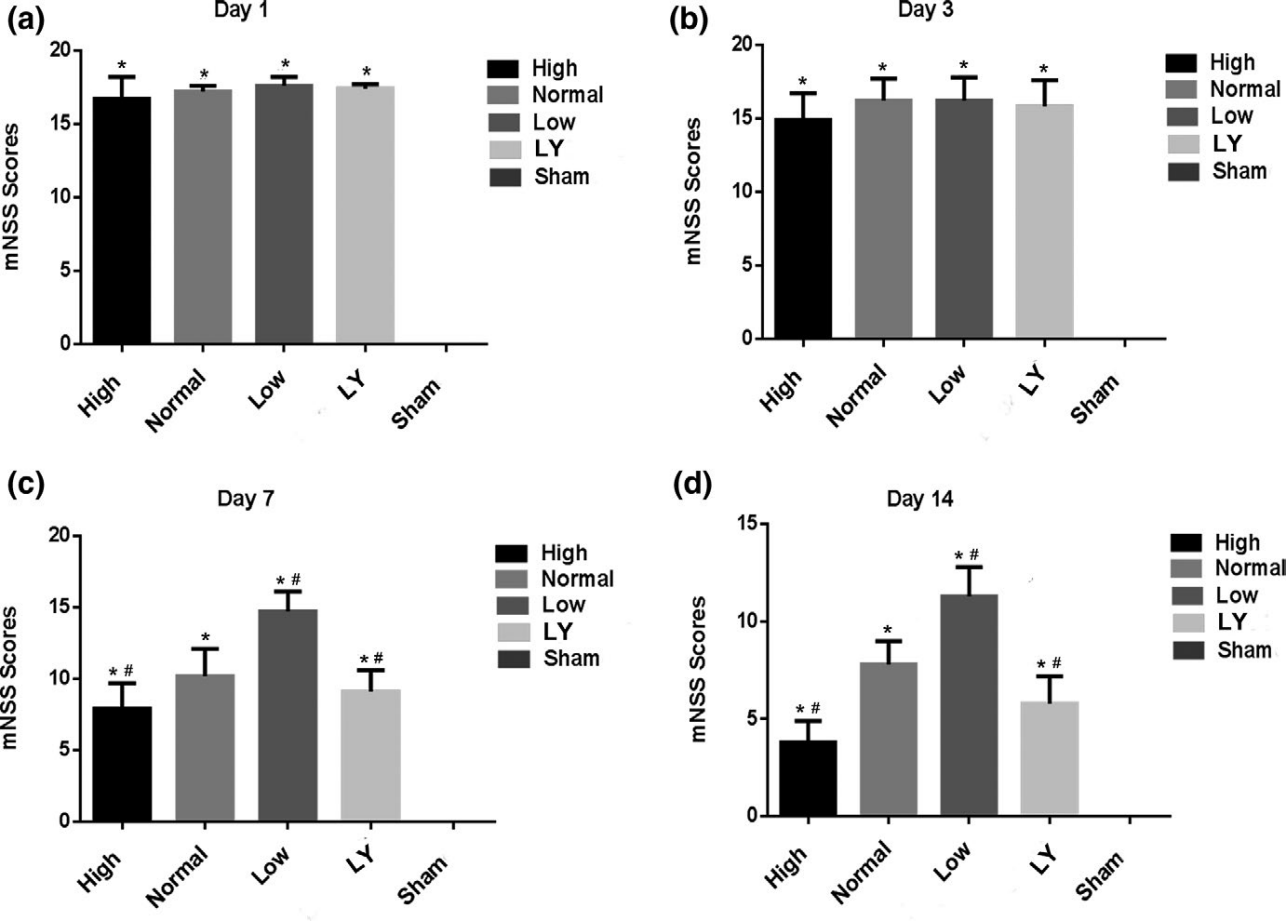
Effects of estrogen on the mNSS in each group. (a) mNSS in each group 1 day after the injury; (b) mNSS in each group 3 days after the injury; (c) mNSS in each group 7 days after the injury; (d) mNSS in each group 14 days after the injury; *p < .05 indicated compared with the Sham group; ^#^ *p < *.05 indicated compared with the normal estrogen group. mNSS, modified neurological severity score. The rats in the Sham group received sham operation of scalp incision as a negative control; in all the other four groups, the rats were received a subcortical axonal fiber injury surgery as the test groups

To determine whether the neuroprotection provided by estrogen was associated with behavioral improvement, motor coordination was evaluated by mNSS and beam walking test. For mNSS as shown in Figure 3, at 1d and 3d after the injury, the mNSS of Sham group was 0, and the other 4 groups scored higher. At 7d, the score of the Sham was still 0, while the other 4 groups were gradually differentiated (*p* < .05). Interestingly, at 14d after injury, the other 4 groups' scores were more and more lower and there was a statistical significance among them (*p* < .05). Furthermore, with the time going on, the mNSS of each group of subcortical axonal fiber injury was lower and lower (*p* < .05).

**Figure 4:**
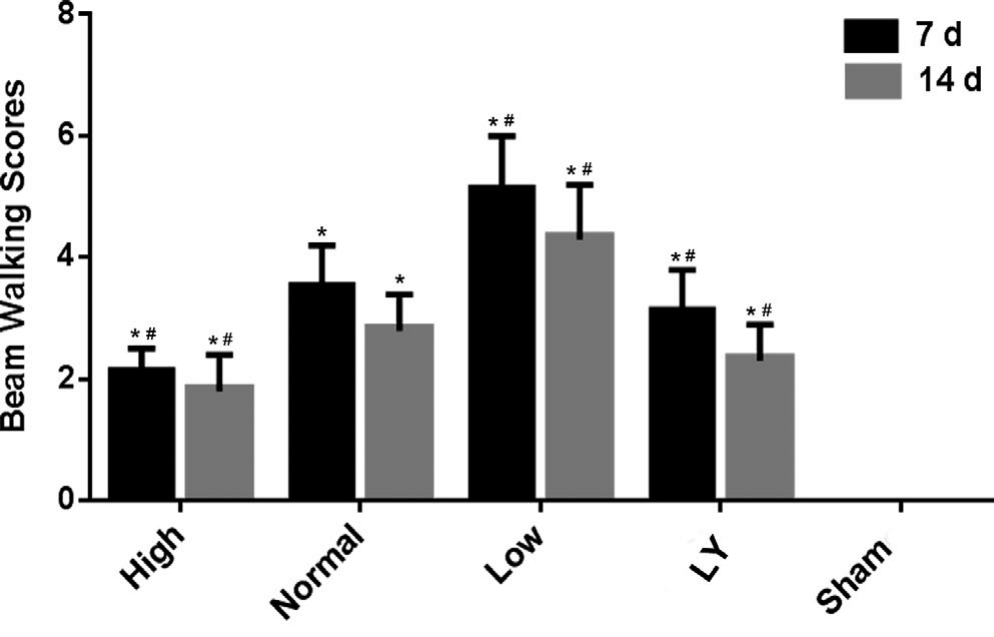
Effects of estrogen on the beam walk scores in each group after estrogen treatment at 7 and 14 days. *p < .05 indicated compared with the Sham group; ^#^ *p < *.05 indicated compared with the normal estrogen group. The rats in the Sham group received sham operation of scalp incision as a negative control; in all the other four groups, the rats were received a subcortical axonal fiber injury surgery as the test groups

The mNSS neurological function score has confirmed that the difference in the nerve function recovery was at 7 days after injury in each group, so, the balance beam test was carried out at 7 and 14 days. At 7 days, the beam working scores of the other 4 groups were all higher in comparison with the Sham group. Among them, high estrogen group scored the lowest, while low estrogen group scored the highest. At day 14, the trend was consistent with that at day 7, and the statistical difference between groups was more significant (*p* < .05; Figure 4).

### 3.3 Postinjury treatment with estrogen alleviated DTI after subcortical axonal fiber injury

**Figure 5:**
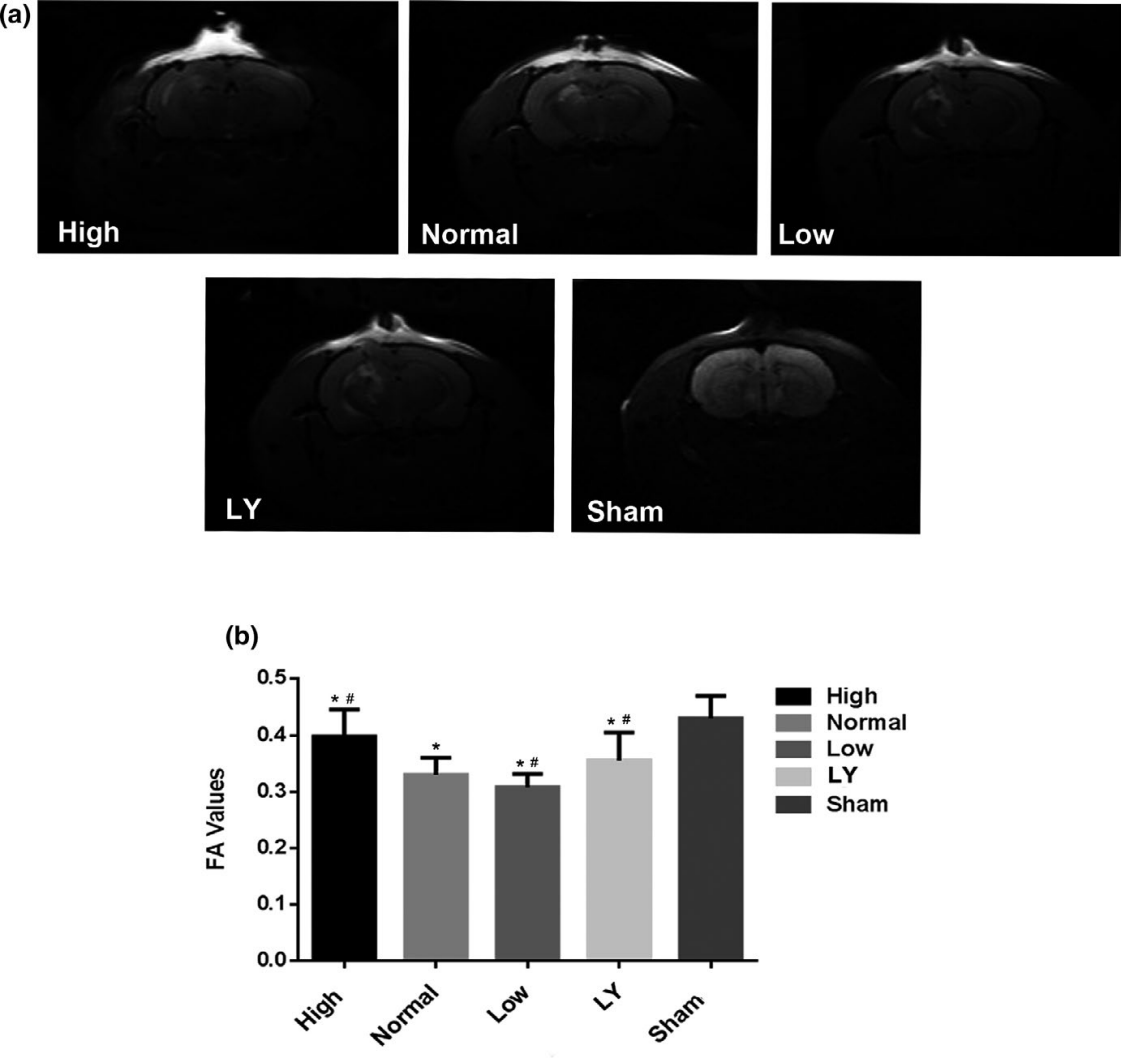
Effects of estrogen on the DTI (FA values) in each group. (a) A DTI scan sequence; (b) FA values in each group; all the data were represented as means ± *SD*; *p < .05 indicated compared with the Sham group; ^#^ *p* < .05 indicated compared with the normal estrogen group. The rats in the Sham group received sham operation of scalp incision as a negative control; in all the other four groups, the rats were received a subcortical axonal fiber injury surgery as the test groups

In Figure 5b, compared with the Sham group, the other four groups were much less, and there was a significant difference between them (*p* < .05). More interesting, even compared to the normal estrogen group, the difference was still statistical (*p* < .05). Figure 5a shows the results of the DTI scan, which can reflect the axon integrity. In addition, we can obtain the FA value by comprehensively analyzing multiple scan sequences.

### 3.4 Effects of estrogen on the expression of Neurofilament 200 after subcortical axonal fiber injury

**Figure 6:**
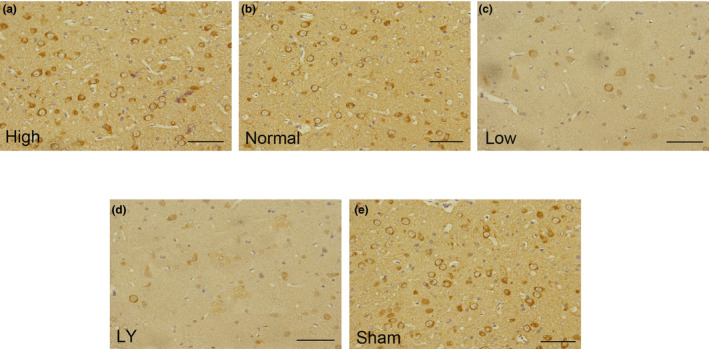
Effects of estrogen on the expression of NF200 in each group. (a) High estrogen group, a strong expression in the membrane of the cells; (b) Normal estrogen group, which has a moderated expression of NF200; (c) Low estrogen group, which has a weak expression of NF200; (d) LY294002 group, after treatment with LY294002, the expression of NF200 decreased significantly; (e) Sham estrogen group, which has a strong expression NF200. Scale bar = 50 µm; NF200, Neurofilament 200; LY, LY294002. The rats in the Sham group received sham operation of scalp incision as a negative control; in all the other four groups, the rats were received a subcortical axonal fiber injury surgery as the test groups

NF200 is a classical marker of the axon regeneration, and it is often expressed on the membrane of the cells. As presented in Figure 6, strong positive expression of NF200 in the membrane of the cells was observed in the Sham group. After subcortical axonal fiber injury and ovariectomy, the expression of NF200 was very weak in each group and the Low group was the weakest. Furthermore, high estrogen treatment had significantly increased the expression of NF200, which was counteracted by LY294002.

### 3.5 Effects of estrogen on the protein expression of p‐Akt, CDK5, p‐Ser262, p‐Ser404, and p‐Thr205 of Tau sites in each group

**Figure 7:**
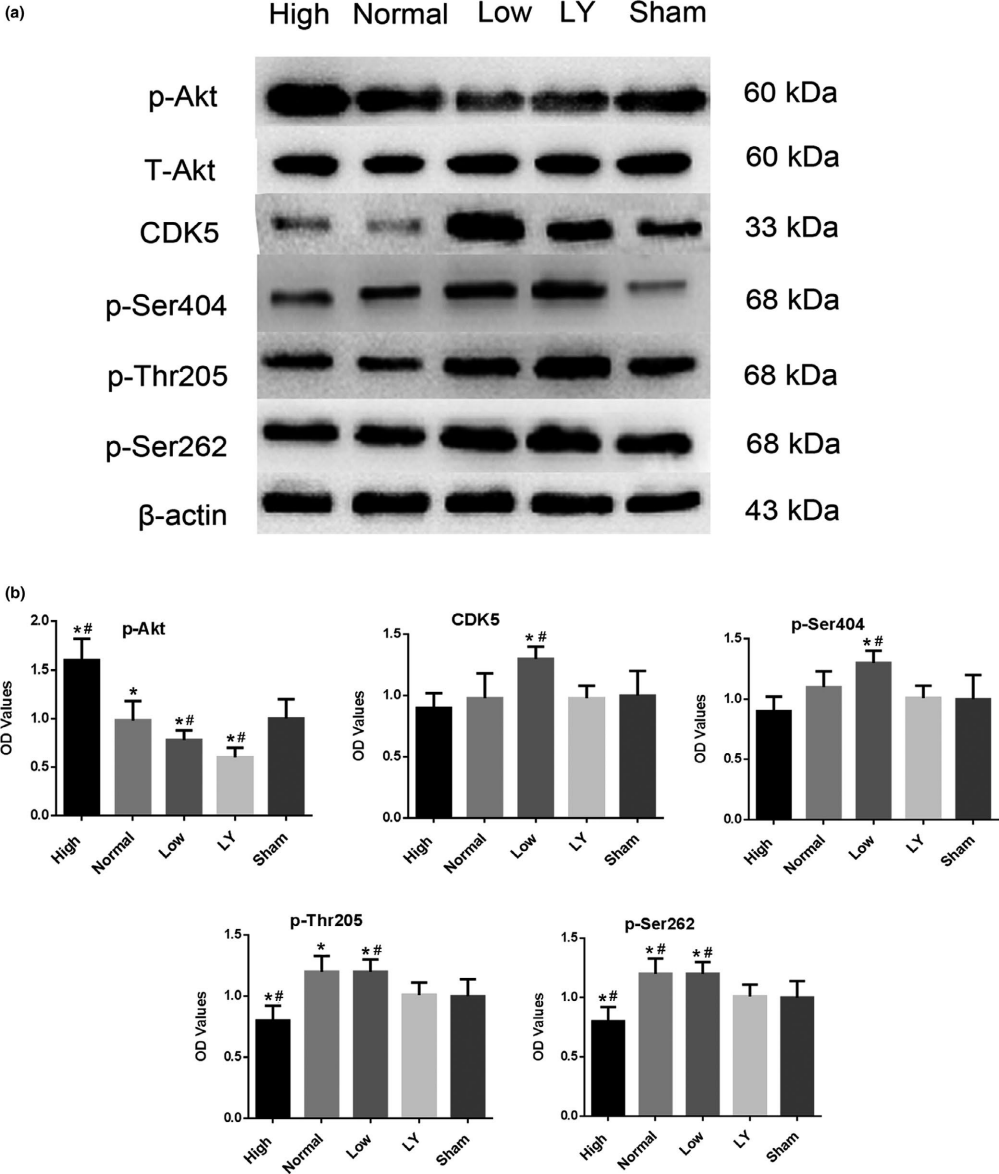
Effects of estrogen on the expression of p‐Akt, CDK5, p‐Ser262, p‐Ser404, and p‐Thr205 in each group. (a) Western blotting results of p‐Akt, CDK5, p‐Ser404, p‐Thr205, and p‐Ser262; (b) The relative optical density (OD) of p‐Akt/T‐Akt, CDK5, p‐Ser404, p‐Thr205, and p‐Ser262, respectively; *p < .05 indicated compared with the Sham group, ^#^ *p* < .05 indicated compared with the normal estrogen group; LY, LY294002. The rats in the Sham group received sham operation of scalp incision as a negative control; in all the other four groups, the rats were received a subcortical axonal fiber injury surgery as the test groups

After binding with its receptor, estrogen can activate PI3K/Akt signaling pathway, and CDK5 is the downstream target gene of the PI3K/Akt pathway. So, in the present study, we detected the expression of Akt and CDK5. As seen in Figure 7, p‐Akt expression was increased after high estrogen treatment, while LY249002 significantly decreased its expression. Compared to the Sham group, the protein expression of CDK5 was much higher in the low estrogen group; however, estrogen treatment significantly decreased its expression, and the addition of LY249002 counteracted the roles of estrogen.

The phosphorylation of Tau at Thr205, Ser262, and Ser404 sites is regulated by CDK5. That is, the higher the expression of CDK5, the higher the phosphorylation level of these three sites. In fact, as shown in Figure 6, p‐Ser404, p‐Ser262, and p‐Thr205 were all significantly higher in the normal estrogen groups in comparison with the Sham group (*p* < .05). After estrogen treatment, their expression was all markedly decreased (*p* < .05). Interestingly, the effects of estrogen on their expression were partly offset by LY 249,002 (*p* < .05).

## 4 DISCUSSION

Estrogens are involved in the development and maintenance of normal reproductive functions. They also play very important roles in the immune system as well as in the central nervous system (CNS) in human body (Chakrabarti et al., 2014). At present, most of the studies were focused on the anti‐apoptotic function of estrogen on neurons, but the effect of estrogen on the regeneration of nerve axons was rarely reported. For example, Kim et al. have shown that estrogen therapy can improve nerve function and repair white matter structure in rats with craniocerebral trauma (Kim et al., 2017). Wang et al. demonstrated the protective effect of estrogen on axonal degeneration (Wang, Zhang, Wang, Qi, & Lou, 2007), while research by Acosta et al. showed that estrogen was necessary for exercise‐mediated enhancement of motoneuron participation after peripheral nerve injury in mice (Acosta, Copley, Harrell, & Wilhelm, 2017). Furthermore, there are many ethical problems in the clinical use of estrogen due to its side effects. Thereby, it is necessary to explore the underlying mechanisms of estrogen for clinical intervention. Herein, we focus on the effect of estrogen on the regeneration of neuronal axons.

It has been reported that estrogen plays a neuroprotective role in CNS diseases (Barbara Mostacci et al. 2018; Sabina Bhatta et al. 2018). Animal experiments also have confirmed that estrogen can improve limb motor function, and there exists sex difference in neuropathology and cognitive behavior in APP/PS1/Tau triple‐transgenic mouse model of Alzheimer's disease (Späni et al. 2018; Raghava et al. 2017; Yang et al. 2018). In our study, we observed that compared with the Sham group, the scores of modified neurological severity and balance beam walking in the rats with subcortical axon fiber injury in each group were increased. In the MWM experiment, the time of escape of the rats was delayed, the number of crossing was reduced after removing the platform, and the time and distance of staying in the quadrant were shortened, which could determine that the modeling of subcortical axon fiber injury successfully caused the neural dysfunction and memory impairment of the rats. Estrogen treatment reduced mNSS, balance beam walking score, and escape time, and improved the number, time, and distance of crossing quadrants.

Neurofilament protein 200 (NF200) is an important protein involved in neuronal regeneration (Yu et al., 2019). To further investigate the effect of estrogen on axonal regeneration, the expression of NF200 was examined. As expected, high estrogen treatment prominently improved its expression. Meanwhile, by ultra‐high magnetic field NMR detection, we found that the FA value of the injured area in the high estrogen group was significantly higher than that in the normal and low‐level groups, indicating that the integrity, parallelism, and density were significantly higher than those in the other groups. The above data showed that the trend of axon regeneration is consistent with the trend of functional recovery, that is, estrogen can restore neural function by promoting axonal regeneration.

It is well known that Tau protein plays an important role in stabilizing microtubule structure, promoting microtubule assembly and dynamically regulating microtubule stability (Lopes et al., 2016). After axon injury, CDK5 is activated, presenting as hyperphosphorylation, thus reduces its ability to bind microtubules, alters the morphology of nerve cells, and leads to skeleton disruption and cell apoptosis (Dhavan & Tsai, 2001). Estrogen binding to receptors can activate PI3K/Akt signal pathway and induce cell proliferation, differentiation, migration, and apoptosis, which has been reported to be involved in various brain injury processes (Tu et al., 2018; Wang, Pan, Xu, & Li, 2017). In the present study, we observed that CDK5, as the downstream substrate of PI3K/Akt signal pathway, was significantly inhibited after high estrogen treatment. Moreover, after the PI3K/Akt pathway inhibitor LY294002 was applied, the expression of CDK5 was restored to the level of normal estrogen level, which indicates that the expression of CDK5 was influenced by the level of estrogen after axonal injury. Moreover, this effect was regulated by estrogen through PI3K/Akt signaling pathway. Furthermore, we also found that p‐Ser404, p‐Thr205, and p‐Ser262 were significantly increased with CDK5 increased and when the expression of CDK5 was inhibited by LY294002, the expression of p‐Ser404, p‐Thr205, and p‐Ser262 was also decreased. All the results suggested that CDK5 directly participated in the phosphorylation process of Ser404 Thr205 and Ser262 sites of Tau protein, which was corresponding with the previous studies (Neri‐Gomez et al., 2017).

In conclusion, estrogen could improve the axonal regeneration and recover the function after subcortical axonal fiber injury, which may be related to the PI3K/Akt/CDK5/Tau pathway.

## CONFLICT OF INTERESTS

The authors declare that they have no conflict of interest.

## AUTHORS' CONTRIBUTION

Xiaohui Xia and Changlong Zhou conceived and designed the study. Xuenong He and Xiaochuan Sun involved in administrative support. Xiaohui Xia, Chang Liu, and Guanyu Wang provided study materials or patients. Chang Liu, Guanyu Wang, and Changlong Zhou collected and assembled the data. Xiaohui Xia and Changlong Zhou analyzed and interpreted the data. All authors wrote and finally approved the manuscript.

## Data Availability

The data used to support the findings of this study are available from the corresponding author upon request.
